# 

**Published:** 1889-08-24

**Authors:** 


### MISS FLORENCE NIGHTINGALE.

### SPECIAL ANNOUNCEMENT.

The portrait of Miss Nightingale is now completed, and will be published in the September Monthly Part of The Hospital; it having been reproduced at great cost and perfection. Every member of the nursing profession will now be able to acquire the likeness of one, who, though raised to pre-eminence among women by a life of self-sacrifice and philanthropic labour, has nevertheless shrunk from the publicity which fame always brings.

The Monthly Number will be issued at 6d., as usual, but orders should be sent in early, as the demand is expected to be very great. Copies of the portrait alone are obtainable at 3d. each. A biographical sketch of Miss Nightingale appeared in The Hospital of July 27th.

### NOTICE TO CORRESPONDENTS.

All MS., Letters, Books for Review, and other matters intended for the Editor, should be addressed The Editor, The Lodge, Porchester Square, London, W.

### Notice to Advertisers and Subscribers.

All Advertisements, Orders for Copies of The Hospital, and Business Communications should be addressed to The Publisher, *not to the Editor*, The Hospital, 139-140, Salisbury-court, Fleet-street, E.C.

Vol. V., now ready, 4s., post free. Cases for binding, may be had of the Publisher, at 1s. 6d. each.

### To Residents, Secretaries, and Matrons.

The Editor will be glad to have the Regulations and each Report as issued by Asylums, Hospitals, Convalescent and Nursing Institutions, as well as those of other Charities, and an early copy in duplicate of all Appeals and Festival Notices, etc., addressed to *The Editor, The Lodge, Porchester-square, London, W.* Any superintendent, secretary, matron, or other chief official who regularly sends such information may be registered, on application to the Publisher, to receive free of cost a cover or binding The Hospital in half-yearly volumes.

### Amusements and Relaxation.

### Second Quarterly Word Competition Commenced July 6th, ends September 28th.

Three Prizes of 15s., 10s., 5s., will be given for the largest number of words derived from the words set for dissection.

Proper names, abbreviations, foreign words, words of less than four letters, and repetitions are barred; plurals, and past and present participles of verbs, are allowed. Nuttall's *Standard* dictionary only to be used.

N.B.—Word dissections must be sent in WEEKLY, arranged alphabetically, with correct total affixed.

The word for dissection for this, the *Eighth* week of the quarter, being

"V E N T N O R."

**Results of Sixth Week.**

| Names. | Aug. 15th. | Totals. | Names. | Aug. 15th. | Totals. |
|---|---|---|---|---|---|
| Nurse Duty | 120 | 416 | Leirion | — | 195 |
| N'importe Qui | 116 | 379 | Matron | — | 174 |
| Jumps | — | 30 | F. C. J. | 91 | 272 |
| Tinie | — | 186 | Percy Verance | — | 18 |
| Patience | 131 | 422 | Gleniffer | — | 175 |
| Amicus | 115 | 394 | Gypsye | 106 | 361 |
| Broxbourne | 112 | 398 | Ladyr | — | 127 |
| Speedwell | 113 | 381 | A. B. | — | 170 |
| Sister | 126 | 401 | Essie | 100 | 326 |
| Lightowlers | — | 292 | W. G. R. | 95 | 322 |
| Lauriston | — | 288 | Cowboy Bill | — | 179 |
| Rattler | — | 28 | M. L. A. | — | 117 |
| Paignton | 104 | 334 | Little Tuppy | — | 110 |
| Fassifern | — | 141 | Embryo | — | 56 |
| W. R. E. | 120 | 395 | K. Jarman | — | 43 |
| Esperance | 131 | 418 | Reveal | — | 196 |
| M. W. | 131 | 423 | Jenny Wren | 99 | 317 |
| Isabel | 116 | 389 | Condorcet | 110 | 346 |
| Buxton | — | 167 | Electrician | 103 | 184 |
| Nux Vom | — | 263 | Pearl | 94 | 132 |
| Qu'appelle | 102 | 359 | R. W. | — | 43 |
| Spes | — | 24 | Nurse Amie | — | 89 |
| Secnarf | — | 229 | Sunshine | 83 | 107 |
| Rover | — | 165 | | | |

**Notice to Correspondents.**

N.B.—All letters referring to this page which do not arrive at 139 and 140, Salisbury-court, London, E.C., by the *first post on Thursdays*, and are not addressed PRIZE EDITOR, will in future be disqualified and disregarded

Competitors can enter for *all* quarterly competitions, but no competitor may take more than *two quarterly prizes* during the year.

**Conditions.**

I. Every paper sent in must be clearly written, and one side only must be used. All competitors must mark at the head of their papers the number of their competition.

II. Each paper must be signed by the author with his or her real name and address. A *nom de plume* may be added if the writer does not desire to be referred to by us by his real name. In the case of all prize-winners, however, the real name and address will be published.

III. All communications relating to prize competitions should be addressed to "The Prize Editor, The Hospital, 139 and 140, Salisbury-court, London, E.C." Competitors are requested not to include in letters, etc., to the Prize Editor communications on matters of other business. All letters must be fully prepaid.

IV. The Prize Editor of The Hospital is the sole judge of the competitions, and his decision is in all cases final and without appeal.

V. The Prize Editor reserves the right to publish any paper sent in, whether it receives a prize or not. He does not hold himself responsible for any MSS. sent, nor can he undertake to return rejected competitions.

### Scraps and Gleanings.

Leeds Public Dispensary treated 4,767 patients last year.

Chippenham Hospital Sunday resulted in a collection of £19.

The death is announced of Dr. Henry Forester, of Barnstaple.

The will of Dr. Charles Elam has been proved; personalty £51,000.

The outbreak of typhoid at the West End has been much exaggerated.

Stockport Provident Dispensary was opened on August 8 by Mr. J. Leigh.

Plans have been accepted for the erection of a new wing to Cardiff Infirmary.

Tea made with boiling milk instead of water is less likely to cause indigestion.

Dr. John Mullen has been appointed medical officer of Wilton Fever Hospital.

The Corporation of the City of London have given £210 to King's College Hospital.

Grimsby District Hospital reports 52 in-patients, and five deaths for the last year.

The Mayor of Bangor (Mr. Peirce) has offered £500 towards an infectious hospital.

Stockton Hospital is going to add three more working men to its General Committee.

Dr. James Russell has been appointed medical officer to Keighley Workhouse Infirmary.

The death is announced of Dr. T. K. Chambers, Honorary Physician to the Prince of Wales.

Dr. Wightwick, of Horselydown, has married Miss Helen D'Alton, the well-known singer.

Mercer's Hospital, Dublin, has received £177 15s. 9d. under the will of the late Mrs. Clouston.

Dr. J. Lloyd Roberts has been appointed medical officer of Colchester Asylum for Idiots.

Messrs. Maple and Co. have sent a cheque for £52 to the North London Consumption Hospital.

The Duke of Connaught has forwarded to the Lord Mayor a cheque for £50 for the Volunteer Patriotic Fund.

The Al Fresco Fete at Margate was well attended and the weather was fine. Captain Blount is to be congratulated.

A valuable silver inkstand has been presented to Mr. Jackson by his colleagues in the Sheffield School of Medicine.

The German Admiralty has given 4,000 marks (nearly £200) towards erecting a second German hospital in Zanzibar.

The Chaplain of Cumberland Infirmary has collected enough money to present a new American organ to the institution.

Dr. J. T. Banks, one of the physicians in ordinary to her Majesty in Ireland, has been made a Knight Commander of the Bath.

The Infectious Hospital, Durham, is to be furnished at a cost of £1,488. The doctor is to be allowed a Brussels carpet in spite of expense.

The Dowager-Countess of Kintore has subscribed £1,000 towards the Jubilee fund which is being raised for the extension of Aberdeen Royal Infirmary.

Lord Bristol's herd of deer at Ickworth Park, Bury St. Edmunds, has been attacked by anthrax. Nearly 200 animals, principally does and fawns, have died.

There are at Constantinople upwards of 250 lepers. Of these 25 individuals only are isolated in special locality at Scutari, the remainder circulating freely in the streets.

Lady Kortright, formerly of Philadelphia, but now living in England, has presented 100,000dols. to the Presbyterian Hospital at Philadelphia to found a Convalescents' Retreat.

The death is announced of Surgeon-Major James Adolphus Smith, which took place on the 3rd inst. at Bhamo, Upper Burmah, of intermittent fever, at the age of forty-one.

German medical journals report the outbreak of a serious epidemic of typhoid fever at Essen. It is stated that some of the medical men have as many as fifty cases under their care.

The Hospital Saturday Fund was first formed in 1874, when it produced only £258. This year its net total product is £5,080, £130 more than was realised last year, and the largest result ever obtained.

The buildings connected with the crematorium at St. John's, Woking, are now completed. The architecture is thirteenth century Gothic, the body of the buildings being in red brick, with Bath stone facings.

At a meeting of the Glasgow Town Council Dr. Russell reported that already 583 deaths from measles had been registered this year, which will be known as the year of the severest epidemic of measles since 1871.

Mrs. North, the wife of a labourer, has given birth to a triplet. Two of the infants had distinct bodies, with perfect heads and arms, but were connected at the abdomen, and there was only one pair of legs. The children were alive.

Madame Katcheff, doctor in medicine, made no small impression at the Paris Hygiene Congress by the appalling picture she drew of the horrors of the sleeping accommodation provided for workmen in Russian factories, which she characterised as exceeding those of steerage passengers in old emigrant ships.

At the Hygienic Congress, Paris, Dr. de Valcour related that a death having taken place from scarlet fever at an hotel in Monaco, the room was relet to another tenant the next day, and he likewise died of the same fever. It was not till a third case of scarlet fever occurred in this room that the hotel was closed and disinfected.

Miss Saleni Armstrong, M.D., will have charge of the Women's Medical Missionary Training School, which is about to be opened by the American Methodists, in Bombay. Dr. Armstrong is a graduate of the Woman's Medical College of Pennsylvania, and she acquired a valuable hospital experience as physician in the New England Hospital in Boston.

